# Enriching Eggs Naturally: The Nutritional Power of Black Soldier Fly Whole Dry Larvae

**DOI:** 10.3390/ani16050774

**Published:** 2026-03-02

**Authors:** Nadya Mincheva, Adelina Petrova, Ivelina Ivanova, Pavlina Hristakieva, Krasimir Velikov, Veselina Panayotova, Diana Dobreva, Tatyana Hristova, Albena Merdzhanova, Katya Peycheva, Rositsa Stancheva, Ivelin Panchev, Atanas Atanassov, Marc Bolard

**Affiliations:** 1 Agricultural Academy, Agricultural Institute, 6000 Stara Zagora, Bulgaria; minchevan@yahoo.bg (N.M.); ivanova24@abv.bg (I.I.); poly_31@abv.bg (P.H.); sherry_bg@abv.bg (K.V.); 2 Center of Competence “Sustainable Utilization of Bio-Resources and Waste of Medicinal and Aromatic Plants for Innovative Bioactive Products” (BIORESOURCES BG), 1000 Sofia, Bulgaria; 3 Department of Chemistry, Medical University of Varna, 9002 Varna, Bulgaria; veselina.ivanova@hotmail.com (V.P.); didobreva@gmail.com (D.D.); tatiana.hristova@mu-varna.bg (T.H.); a.merdzhanova@gmail.com (A.M.); peytcheva@hotmail.com (K.P.); rositsa.stancheva@mu-varna.bg (R.S.); 4 Joint Genomic Center, Sofia University, Dragan Tzankov 8, 1164 Sofia, Bulgaria; ipanchev@abv.bg (I.P.); atanas_atanassov@jgc-bg.org (A.A.); 5 Nasekomo EAD, Saedinenie 299, 1551 Lozen, Bulgaria; marc.bolard@nasekomo.life

**Keywords:** black soldier fly, carotenoids, antioxidants, vitamins, fatty acids, hens’ diet, egg enrichment

## Abstract

The current investigation examined the effect of inclusion of Black soldier fly (*Hermetia illucens*; BSF) dry larvae in hens’ diet on egg quality and hen performance. A total of 260 brown egg-laying hens (RIR × RIW) were divided into four groups (65 hens/group; 5 pens/group) and fed with control and experimental diets (inclusion rates 3%, 6%, 9%). Although the four-week feeding period did not influence production parameters, yolk color responded positively to the test diets, showing a linear increase with the percentage of BSF inclusion rate (*p* < 0.001). This was reaffirmed by the increased levels of yolk carotenoids (astaxanthin and β-carotene) and α-tocopherol, with notable differences in 6% and 9% BSF-fed groups (*p* ≤ 0.003). Lipids are an important factor in carotenoid absorption and assimilation, and the combination of fat content and carotenoids in BSF suggests the potential of this system for egg enrichment. Along with the increased antioxidant levels, a novel finding is the positive correlation between BSF inclusion rates and hens’ egg yolk levels of C:15 and C:17 fatty acids, key players in the core mechanisms of cell health and longevity. Altogether, the results provide evidence of the potential of BSF for enrichment of eggs with carotenoids and vitamins with strong antioxidant activity, which would have a positive effect on human health.

## Simple Summary

Black soldier fly larvae (*Hermetia illucens*; BSF) are one of the key insects subjected to commercial farming world-wide. BSF has excellent nutritional parameters, which underlines its potential for the poultry industry. Along with their nutrition profile, BSF larvae carry many biologically active molecules that may positively affect animal health and products. Importantly, tailor-made diets can produce BSF products with different chemical profiles to target specific aims in animal feeding. To elucidate one of these aspects, the study tested the effects of inclusion of alfalfa-fed BSF whole dry larvae in hen’s diet at different concentrations (3%, 6%, 9%), on egg quality and hen’s performance. Although the four-week feeding period did not influence production parameters, BSF-fed hens produced eggs with enhanced yolk color and increased levels of carotenoid and vitamin content (compared to hens fed on regular diet with no BSF), which are characterized with high antioxidant activity. Furthermore, results in this study suggest that BSF-fed hens produced eggs with different fatty acid profiles that might be beneficial for human health. Altogether, results suggest that biologically active components of BSF might be important factors in animal feeding to improve health, performance, and product quality with possible improvement of human health.

## 1. Introduction

Eggs are an excellent source of macro- and micro-nutrients, with relatively low caloric value (140 kcal/100 g), low economic cost and broad culinary potential. The new consumer appreciation of eggs is based not only on their balanced and rich nutrient profile (rich in proteins, essential fatty acids, vitamins, minerals), but also on their potential for health-promoting biological activities. Many egg proteins and peptides have been shown to have antimicrobial, antioxidant, and anti-cancer properties [1,2,3]. The egg yolk is a good source of vitamins (A, D, E, K, B1, B2, B5, B6, B9, and B12) and essential fatty acids (FA). Eggs are rich in choline (in the yolk, 680 mg/100 g), an essential nutrient with diverse biological functions, including neurotransmission and brain development [4,5,6]. Carotenoids are another group of bioactive compounds that are present in the egg yolk. Hens cannot synthesize carotenoids de novo, which underlines the importance of the carotenoid level in the diet. Carotenoids have strong antioxidant activity, and based on their chemical structure, are classified into two groups: carotenes and xanthophylls [7,8]. Carotenes are conjugated C40 hydrocarbon chains that may form a linear (lycopene) or cyclic structure (b-carotene, a-carotene). The second group is that of xanthophylls, which are oxygenated carotenes. Upon ingestion, carotenoids are released from the food matrix by the digestive enzymes and are solubilized into mixed micelles consisting of FA, phospholipids, bile acids, cholesterol, and monoacylglycerols to enable intestinal absorption [9]. Thus, lipids are an important factor in carotenoid absorption and assimilation. Two uptake mechanisms of carotenoids in the intestine are known: through passive diffusion and through active receptor-mediated transport. Following intestinal absorption, carotenoids are packed into lipid-rich particles to be delivered to the liver [10,11]. In the liver, carotenoids are processed and integrated into low-density lipoproteins (LDL), high-density lipoproteins (HDL), and very low-density lipoproteins (VLDL) for transportation to different tissues via the bloodstream [12,13]. In the ovaries, lipids and carotenoids are delivered by VLDL for the processes of oogenesis and formation of the egg yolk [12,14]. Thus, carotenoids in hens’ diet, once ingested, go through complex processes before impacting the egg yolk; this understanding can help to enhance the effectiveness of egg-enrichment methods.

In hens’ diets, the main sources of carotenoids (from the usual components used in the diet) are corn, green forages (alfalfa, clover), and peas. Since the consumers’ perception of healthy eggs is associated with yolk color, different strategies for carotenoid enrichments are used, either through natural or synthetic compounds. In modern poultry farming, often synthetic carotenoids are used as feed supplements to enhance yolk color. While canthaxanthin is preferred for red, the β-apo-8’-carotenoic acid ethyl ester is preferred for yellow; both are used under different commercial names. Synthetic carotenoids are effective in enhancing yolk color; however, there is public concern regarding the effects of these additives, since some animal studies have reported their accumulation in the retina as crystalline deposits [15]. As an alternative, natural sources of carotenoids were tested by supplementing hens’ diets. Many plant products such as red pepper powder, tomato by-products, alfalfa, and extracts from marigold and calendula included into hens’ diets have a positive effect not only on yolk color, but also on egg quality and hens’ performance [16,17].

Carotenoids are potent antioxidants, and when ingested, their biological impact is translated into anti-inflammatory, immunomodulatory, and anti-cancer properties, with a number of human health benefits related to the prevention of eye diseases (cataracts, macular degeneration), certain types of cancer, cardiovascular and skin diseases, neurodegenerative disorders [18,19,20,21,22].

The use of insects in hens’ diets has the potential to increase the sustainability of egg production [23]. Black soldier fly (BSF; *Hermetia illucens*) larvae have a special status among other farmed insect species due to their unique capabilities of bioconversion of a wide range of organic wastes [24,25]. Importantly, BSF larvae can accumulate certain biologically active compounds present in their feed, like, for instance, omega-3 fatty acids [26,27]. BSF larvae are rich in saturated fatty acids (SFA) but low in polyunsaturated fatty acids (PUFA), especially omega-3. Several studies, however, demonstrated that inclusion of feed ingredients rich in omega-3 in larval diets results in increased PUFA levels in the larvae [26,27]. Other investigations reported successful production of carotenoid-enriched BSF larvae [28,29,30] when reared on diets containing agro-food by-products (tomatoes, carrots, kale, parsley, broccoli, endive), which is a sustainable strategy to recycle some carotenoids and vitamins back into the food cycle [28]. Overall, such enrichment of BSF larvae through diet manipulation is a powerful tool to create tailor-made products so as to meet specific requirements of animal nutrition. However, to achieve the desired effects in animals (product quality, performance, health) following feeding with BSF product, several other factors must be considered, such as type of BSF product (defatted vs. whole dry larvae vs. live larvae), dose, exposure, etc. Previous investigations from our group on feeding hens with BSF live larvae recorded a negative impact on yolk color [31]. Yolk color from BSF-fed hens had a paler color compared to that of the control group. To address this problem, the present study tested the effect of inclusion of BSF whole dry larvae, whose diet contained 5% alfalfa, on hens’ performance and egg quality. To understand the impact of this product, and, in particular, the quality of egg yolk, the study investigated inclusion rates of 3%, 6%, and 9%. Along with assessment of hens’ performance parameters, morphological and chemical analyses were carried out to evaluate egg quality.

## 2. Materials and Methods

### 2.1. Animal Care

The experimental procedures were approved by the Commission on Animal Ethics of the Bulgarian Food Safety Agency to the Ministry of Agriculture and Food (Protocol No. 298/2021).

### 2.2. Experimental Design

The experiment was carried out at the Agricultural Institute, Stara Zagora, Bulgaria, using brown egg-laying hens, crosses between Line T (Rhode Island Red; RIR) and Line N (Rhode Island White; RIW) from the national gene pool. To achieve a uniform laying rate between groups, egg production was recorded daily for 1 month prior to the trial so that groups were comparable at the start of the trial. A total of 260 hens (51 weeks old), with an average weight of 1968.50 ± 36.89 g and egg production rate of 85.38 ± 6.34%, were divided randomly into 4 groups (control, 3, 6, 9% BSF), with no significant differences in live weight and egg production rate between the groups. Each group had 65 hens, which were housed into 5 pens with 13 hens/pen. Thus, 20 pens with the required equipment for hens’ floor rearing system—common trough feeder (10 cm feeding front), drinker (2.5 cm drinking front), laying nests (1 nest/5–6 hens), and perches (15 cm/hen)—were used for the whole experiment. Temperature (23 °C to 26 °C) and humidity (50% to 62%) were monitored daily, and the hens were reared under a 16/8 h light/dark period. Food and water were given ad libitum. One week prior to the beginning of the experiment, all groups of hens were fed with the control diet to enable an equal start.

#### Experimental Diets

BSF whole dry larvae were obtained from “NASEKOMO” EAD. On a specific request for this experiment, the larvae were fed with a standard “Nasekomo” diet (wheat bran and spent yeast) with inclusion of 5% alfalfa. Analyses of the proximate composition of BSF larvae and feed (Table 1) were carried out at the Feba lab, Sofia, Bulgaria, using standard protocols accredited by the Bulgarian Institute for Standardization. Crude protein was determined by the Kjeldahl method (protocol BDS-11374, t.4). Total lipid content was extracted using the Soxhlet method (protocol BDS11374, t.4.4). Analyses for carbohydrates, sugar, fibers, and ash were carried out following protocols RPK 7.2-1:200, BLM 86:2014, BDS 11374 t. 4.3/BLM83:2014, and BDS 11374 t. 4.5, respectively. Both sodium and calcium were estimated by protocol BDS EN ISO 6869 [32]. For determination of dry matter content, protocol BDS 11374 t. 4.1 was used. Energy calculations were carried out using methods RPK 7.2-1:2020/BLM 81:2014/BLM 82:2014.

Four isonitrogenous and isocaloric diets were specially formulated and produced for this experiment. The ingredients and nutrient composition of the diets are shown in Table 2. For the control diet, cereals and soybean meal were used, while in the experimental diets, portions of the conventional protein and fat sources were replaced with increasing levels of 3%, 6%, and 9% BSF dry larvae. All diets were formulated to meet nutrient recommendations of the National Research Council [33], following standard feeding protocols tailored to the nutritional needs of laying hens. No synthetic pigments were added to the feeds, and the level of maize inclusion was the same for all groups (38%) to minimize its influence on yolk coloration. BSF dry larvae were manually crushed into small pieces (~4 mm) and then mixed into the feed with a concrete mixer at the appropriate doses to achieve inclusion rates of 3%, 6%, and 9%.

### 2.3. Determination of Productive Performance

During the experimental period, hens appeared to be in good health, as daily observation recorded no occurrence of dead or ill hens. Eggs were collected and recorded daily. Egg production was expressed as hen-day egg production (%), calculated by dividing the total number of eggs collected by the total number of “hen-days”. Eggs were weighed, and the average egg weight was calculated. Egg mass (g/hen/day) was then determined by multiplying the average egg weight by the hen-day egg production (%). Feed intake was analyzed weekly by recording the amount of feed offered and subtracting the feed refusals for each replicate pen. Data on feed intake and egg mass were used to calculate the feed conversion ratio (feed intake/egg mass; g/g). All performance parameters were calculated for each of the five replicates per group and summarized over the 4-week experimental period.

### 2.4. Determination of External and Internal Egg Quality

At the end of the experiment (55 weeks of age), eggs laid on the sampling day were collected from each pen. A total of 50 eggs per treatment (10 eggs/pen) were randomly selected and analyzed within 24 h after collection for egg quality parameters. The egg shape index was calculated based on the measurements of the egg width and length (digital caliper). Egg weight, yolk color intensity, and Haugh units were measured using the Egg Multi-Tester EMT-5200 (Robotmation, Co., Ltd., Shibuya, Tokyo, Japan). Yolk and egg shell weight measurements were made with an electronic balance (MVP-300 H). Albumen weight was determined by subtracting yolk and shell weights from the respective egg weight. The egg shells were washed with tap water, air dried at room temperature, and then weighed. Values obtained from the measurement of the weight of egg components (yolk, albumen, shell) were used to calculate the proportion of these components. Egg shell thickness was evaluated by taking the mean of three measurements (at both ends and the equator) using a screw thread micrometer. For analyses of FA, carotenoids, and vitamin compounds, 50 eggs from each group were collected (at the end of the feeding trial) at random.

### 2.5. Chemical Analyses

#### 2.5.1. Chemicals

All standards (all-trans-retinol, α-tocopherol, β-carotene, astaxanthin, fatty acid methyl esters) were purchased from Merck KGaA (Darmstadt, Germany). All solvents used (chloroform, methanol, dichloromethane, and n-hexane) were of HPLC grade and purchased from Sigma-Aldrich (Burlington, MA, USA).

#### 2.5.2. Fatty Acid Composition

Lipids from BSF larvae and egg yolks were extracted following the Bligh and Dyer procedure [34], as previously described [35]. Three replicates of black soldier fly larvae (20 g each) were homogenized with a laboratory blender (Isolab Laborgeräte GmbH Co., Eschau, Germany). Briefly, larvae and egg yolk homogenates (1 g) were subjected to subsequent extraction steps with chloroform/methanol (1:2 *v*/*v*), chloroform/methanol (1:1 *v*/*v*), and chloroform. Methyl esters of the lipids were prepared using direct transmethylation with 2% sulfuric acid in methanol [36]. The organic layer was taken for FA (FAMEs) analysis using a Thermo Fisher Scientific FOCUS—PolarisQ Ion Trap GC/MS (Thermo Fisher Scientific, Waltham, MA, USA). Each extract (1 μL), containing the total FAMEs, was injected into a capillary column (Trace™ TR-FAME, Thermo Fisher Scientific, Waltham, MA, USA; 60 m × 0.25 mm × 0.25 μm) with a split ratio of 10:1. Helium was used as a carrier gas at a flow rate of 1.2 mL/min. The oven temperature was programmed from an initial oven temperature of 100 °C for 1 min, followed by a rate of 10 °C/min from 100 °C to 160 °C, raised at a rate of 5 °C/min from 160 °C to 215 °C held for 6 min, and next at a rate of 5 °C/min from 215 °C to 230 °C, which was held for 5 min. FAMEs peaks were identified based on the comparison of the retention times with the authentic standards (Supelco 37 Component FAME Mix). The results were expressed as weight % of total FA.

#### 2.5.3. Fat-Soluble Vitamins and Carotenoids

The sample preparation procedure was performed using the modified method of Dobreva [37]. Briefly, an aliquot of the homogenized sample (0.500 ± 0.005 g) was weighed into a glass tube with a screw cap, and 1% of methanolic L-ascorbic acid and 0.5M methanolic potassium hydroxide were added. Three parallel tests were prepared and subjected to saponification at 70 °C for 30 min. After cooling, samples (all-trans-retinol, α-tocopherol, β-carotene, and astaxanthin) were extracted two times with n-hexane:dichloromethane = 2:1 (*v*/*v*) solution. The combined extracts were evaporated and re-dissolved in methanol:dichloromethane solution, filtrated (0.45 µm syringe filter), and injected (20 μL) into the HPLC system. The chromatographic analysis was performed on the HPLC system (Thermo Fisher Scientific, Waltham, MA, USA) equipped with UV2000 and FL3000 detectors. All-trans-retinol, α-tocopherol, β-carotene, and astaxanthin were determined simultaneously using a reverse-phase analytical column (Synergi 4μ Hydro-RP 80A pore 250 mm × 4.6 mm), and a mobile phase 75:20:5 = acetonitrile: methanol: 2-propanol (ACN: MeOH: iPrOH), at 1.1 mL/min. Detection of β-carotene (λ = 450 nm) and astaxanthin (λ = 474 nm) was performed with a UV detector. Concentrations of all-trans-retinol (at λex = 334 nm and λem = 460 nm) and α-tocopherol (at λex = 288 nm and λem = 332 nm) were measured by fluorescence detection.

### 2.6. Statistical Analysis

The experiment is based on the principle of randomized complete block design. All the data sets were analyzed using the SPSS software (ver.19.0, IBM Corp, Armonk, NY, USA). A general linear model with repeated measures was used for significance analysis of layers’ performance among different diets and weeks. The diet was set as a between-subject factor, while week was set as a within-subject factor. The post hoc Tukey test was employed to gauge differences between the treatments. The one-way ANOVA model was used for analysis of egg quality parameters (external and internal characteristics of eggs, fatty acids, and antioxidant compounds in egg yolk). Orthogonal polynomial contrasts were applied to test the linear and quadratic effects of the increasing levels of dietary BSF larvae. Replicates were the experimental units. Significant differences were set at *p* < 0.05, and the trend to difference was set at *p* < 0.10.

## 3. Results

### 3.1. BSF Whole Dry Larvae Carotenoids, Vitamins, and Fatty Acid Profile

**Figure 1:**
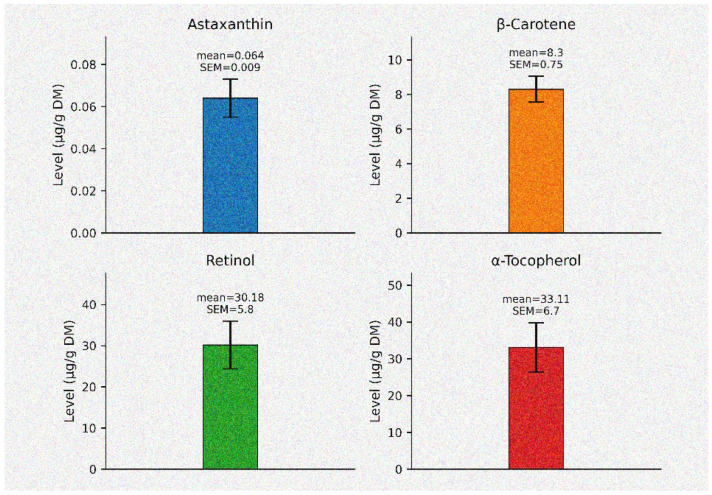
Carotenoid and vitamin levels (µg/g DM) in BSF whole dry larvae (n = 3, SEM±).

BSF whole dry larvae were subjected to analyses in order to establish the levels of carotenoids (β-carotene and astaxanthin) and vitamins (retinol and α-tocopherol). All four parameters were present in BSF whole dry larvae (Figure 1). Compared to the other three parameters, astaxanthin levels were the lowest, at 0.064 µg/gr DM. In contrast, the retinol and α-tocopherol had a more noticeable presence, at 30.18 µg/gr (DM) and 33.11 µg/gr (DM), respectively.

The FA profile of BSF whole dry larvae, shown in Table 3, indicated that the group of saturated fatty acids (SFA) had the highest percentage (72.83% from total FA), while monounsaturated fatty acids (MUFAs) and polyunsaturated fatty acids (PUFAs) were 19.67% and 7.5%, respectively. Among the FAs, the lauric acid (C12:0), palmitic acid (C16:0), and oleic acid (C18:1 n-9) were the most present, at 39.2%, 15.56%, and 15.34% respectively. The majority of analyzed PUFAs were below the detection limits or negligible amounts; however, the most noticeable result was that of linoleic acid (C:18:2 n-6), at 6.4% (Table 3).

### 3.2. Hens’ Productive Performance

Dietary inclusion levels of BSF whole dry larvae affected hens’ feed intake (*p* = 0.004) but had no effect on egg production, feed conversion ratio (FCR), egg weight, or egg mass (Table 4). Over the four-week period, the average values of feed intake decreased linearly, corresponding to the increasing percentage of BSF inclusion in the diet (*p* < 0.001). The average egg production rates varied between 82 and 84% among all groups, with no significant differences between control and experimental groups (*p* = 0.723). The FCR is an important marker in measuring the potential of feed for conversion into egg mass. The average values for the experimental period varied between 2.33 and 2.43, with no significant differences between the control and experimental groups (*p* = 0.429). The average values of the other two performance parameters—egg weight and egg mass—also showed no significant effects from the experimental diets (*p* = 0.465).

### 3.3. External and Internal Egg Quality Parameters

Egg quality is defined by several external (egg weight, shape index, etc.) and internal (albumen, yolk) parameters, which were measured over the experimental period and analyzed (Table 5). While the majority of parameters showed no significant differences, yolk color, however, responded positively to the experimental diets, showing a linear increase with the percentage of BSF inclusion in the diet (Table 5; *p* < 0.001). It is important to note that the differences in egg yolk color units from the 6% and 9% BSF groups (7.24 and 7.45, respectively; *p* < 0.001) were statistically significant from that of the control. A slight decrease in Haugh units in the 9% BSF group was noted, although this was on the borderline of statistical significance (Table 5; *p* = 0.054). In the same group (9% BSF), an increase in shell thickness was also observed (Table 5; *p* = 0.008).

### 3.4. Analysis of Egg Yolk Carotenoids, Vitamins, and Fatty Acid Profile

**Figure 2:**
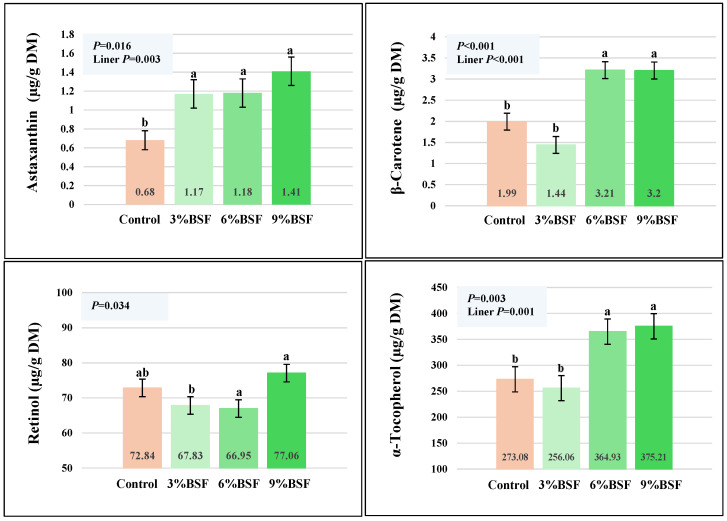
Carotenoids and vitamins from egg yolks (DM) of hens fed with no inclusion of BSF (Control) and with BSF whole dry larvae at 3%, 6%, and 9% inclusion rates. Values with different superscripts (^a,b^) are significantly different *p* < 0.05; (SEM±, n = 3).

To establish the effects of BSF inclusion in hens’ diets, egg yolks from the control and experimental groups were analyzed for carotenoids, vitamins, and FA. Following measurements, analyses showed an increase in carotenoids in the yolks of the BSF-fed groups of hens. However, while significant differences for astaxanthin were noted in all experimental groups (1.17–1.41 µg/g) compared to the control (0.68 µg/g; *p* = 0.016), β-carotene levels responded in the 6% BSF-fed and 9% BSF-fed groups (~3–3.2 µg/g vs. control 1.99 µg/g; Figure 2) and not the 3% BSF-fed group. Both parameters had significant linear responses to the rate of BSF inclusion (Figure 2). Although no significant differences in yolk retinol (Vitamin A) levels were observed in the experimental groups compared to the control, significant differences were observed between the 9% BSF-fed and 3% BSF-fed groups (*p =* 0.034; Figure 2). In the case of yolk α-tocopherol (Vitamin E), significantly higher levels were observed in the 6% and 9% BSF-fed groups (364.93 µg/g and 375.21 µg/g DM, respectively) compared to the control (273.08 µg/g; *p* = 0.003; Figure 2) and the 3% BSF-fed group. BSF-based feed had some influence on the overall yolk FA profile (Table 6), albeit not drastic. The experimental diets did not impact the total SFA levels, although a linear correlation was observed (Table 6; *p* = 0.043). Significant linear increases were noted for the myristic acid (C14:0; *p* = 0.001) and stearic acid (C18:0; *p* = 0.005) levels. An important observation was made regarding the responses of the yolk odd-chain FAs pentadecanoic acid (C15:0) and heptadecanoic acid (C:17) to the BSF-based diets. It must be noted that these were found in BSF larvae at levels of ~1–1.4% of the total FA (Table 3). The total levels of MUFAs and PUFAs were similar between the control and BSF-fed groups, although small statistical differences were noted for myristoleic acid (C14:1; *p* = 0.020), eicosenoic acid (C20:1; *p* = 0.039), and arachidonic acid (C20:4 n-6; *p* = 0.029), with a linear increase related to the rate of BSF inclusion.

## 4. Discussion

Many investigations have reported hens’ responses to BSF products [38,39,40,41]. The fact, however, that both nutritional and bioactive profiles of BSF [42] can be altered through diet composition presents practically limitless possibilities for generating different products that can address specific animal needs. Thus, very detailed research investigations are required to unlock the potential of BSF products so as to promote sustainability in poultry, and importantly, to improve product quality. The focus of this study was testing the hypothesis that incorporation of BSF whole dry larvae (fed on a diet containing 5% alfalfa) into hens’ diet can alter hens’ performance and the antioxidant status of eggs. Testing different inclusion rates of 3%, 6%, and 9% enabled gaining a better understanding of BSF effects, and importantly, identifying dose–response trends, thus strengthening the validity of the results. To assess the differential responses, control and treatment groups of hens received feeds that were isocaloric and isonitrogenous. Furthermore, diets of each group (control, 3% BSF, 6% BSF, 9% BSF) contained equal amounts of maize (38%) to minimize its influence on yolk coloration and antioxidant levels (Table 2). While the four-week BSF feeding trial had no impactful effect on hens’ performance (Table 4), yolk color (*p* < 0.001; Table 5) and yolk antioxidant levels (Figure 2) did respond to the treatment diets. Eggs from the control group had paler yolk color (5.88 units) when compared to the group of 6% and 9% BSF-fed hens that produced eggs with significantly higher levels of pigmentation, at 7.24 and 7.45 units (*p* = 0.000; Table 5), respectively. This suggests that the 4-week feeding period was sufficient for the transfer of carotenoids from BSF whole dry larvae to the egg yolk when hens were fed with higher doses, but not with the lower dose (3% BSF-fed group—6.83 units). Furthermore, these findings indicate that BSF whole dry larvae have ample amount of carotenoids to be absorbed and transferred into the egg yolk. The mechanisms of absorption and transfer are discussed in the Introduction, but the importance of lipid-rich particles acting as vesicles for carotenoid absorption and transfer must be underlined [10,11,12,13,14]. BSF whole dry larvae have high lipid content, plus the propensity to accumulate compounds from the diet, including carotenoids [28,29,30]. These factors emphasize the potential and significance of this feed for poultry. To reaffirm the above results, egg yolks from the control and treatment groups were analyzed (Figure 2) for the same carotenoid (astaxanthin, β-carotene) and vitamin (retinol, α-tocopherol) parameters that were determined in BSF larvae (Figure 1). Measurements of yolk carotenoids showed a positive response to the BSF-based diets (Figure 2), but with different response levels. All experimental groups had higher levels of astaxanthin (1.17, 1.18, 1.47 μg/g, DM for 3–6–9% BSF; Figure 2) compared to the control (0.68 μg/g DM; *p* = 0.016), with a positive linear correlation (*p* = 0.003). The fact that eggs from all treatment groups had significant increases suggests high effectiveness of the transfer, which includes the processes of absorption, transport, metabolism, and deposition. Astaxanthin is a part of the xanthophyll sub-group of carotenoids with very powerful antioxidant activity that translates into wide-ranging health-promoting benefits such as anti-inflammatory, anti-proliferative, and anti-apoptotic effects [43,44]. In poultry, supplementation with astaxanthin resulted in positive effects on hens’ health and egg quality [45]. However, recent studies have shown that higher doses of astaxanthin may alter egg nutrition by decreasing vitamin content and modifying the FA profile [46]. Thus, detailed investigations are required to establish effective doses for the fortification of eggs with this compound. In contrast to astaxanthin, a significant increase (60%) of β-carotene was found in the 6% and 9% BSF-fed groups (*p* < 0.001; Figure 2) but not in the 3% BSF-fed group. Taken together, these two independent analyses (yolk color and carotenoid levels) reinforce the credibility of the above results and demonstrated that inclusion of alfalfa-fed BSF whole dry larvae in hens’ diets has the potential to alter the carotenoid level in eggs. Furthermore, data suggest that each type of carotenoid has different and specific dynamics of transfer that must be studied in detail. The results align with a previous study, where inclusion of BSF meal in hens’ diet resulted in carotenoid enrichment of eggs [47], although the higher level of corn (compared to the control) in the treatment diet may have skewed the results to some extent. To date, several feeding trials with BSF products have reported both positive [48,49] and negative results for the effects on yolk color [31], underscoring the need to examine the influence of multiple contributing factors such as the type of BSF product and its preparation, inclusion rates, and BSF diet-mediated alterations.

Further analyses of the egg yolks from the control and BSF-fed group were carried out to measure retinol (vitamin A) and α-tocopherol responses (Vitamin E). While the retinol levels in the treatment groups were not affected by the BSF-based diets, the α-tocopherol levels showed a positive and significant response. The 6% and 9% BSF-based diets enriched the egg yolk with 364.93 µg/g (DM) and 375 µg/g α-tocopherol, respectively, while in the control, the corresponding level was 273.08 µg/g (*p* = 0.003; Figure 2). This 33–37% range of increase is close to results from previous studies on BSF feeding trials, reporting increases between 25 and 66% for yolk tocopherols as a result of BSF product inclusion [47,50].

The next phase of investigation focused on understanding how the inclusion of BSF whole dry larvae in hens’ diets affected the yolk FA profile, a core factor of egg nutritional value. Yolk FAs responded to hens’ diet components [51], and in this study, results also suggested some impact. While the overall levels of SFAs were not affected by the experimental diets, individual FAs responded. Egg yolks from the 6% and 9% BSF-fed hens had a 44% and 71% increase in myristic acid (C14:0; *p* = 0.004), respectively (Table 6). Some elevations were also noted for stearic acid (C18:0; *p* = 0.016) as well. Other studies also reported a significant transfer of myristic acid into the egg yolks of BSF-fed hens [52]. Furthermore, similarly to our results, the study also noted no accumulation of lauric acid (C:12) in the yolks of BSF-fed hens, as its presence in BSF dry larvae is significant, accounting for almost 40% of the total FA. Increased levels of other SFAs (C15:0, C17:0, C20:0) were noted, although differences from the control group were on the borderline of statistically significant levels (*p* = 0.07–0.08; Table 6). In the past, SFAs were regarded as cholesterol-raising compounds. Current advances in research, however, are changing our perception of this model, as many SFAs are essential elements for cell structure and function, and their biological role and impact are a result of interactions with other FAs or other compounds [53,54]. For instance, while C14:0 increases plasma cholesterol, C18:2 lowers it [53,54]. Thus, current interpretations of FA results must focus on understanding the complexity of FA interactions that dictate the biological impact in the cell. Of particular interest are the odd-chain FAs C15:0 and C17:0 (pentadecanoic and heptadecanoic acid, respectively), as recent investigations underscored their significant role in cell health and longevity [55,56]. The “French paradox” is starting to be associated with these as well, as some full-fat dairy products have the most C15:0. In the case of the current study, all experimental groups had higher levels of C15:0 and C17:0 (compared to the control), although results were on the borderline of statistical significance (*p* = 0.07–0.08; Table 6). The significant linear correlation (*p* = 0.022–0.018; Table 6), however, between BSF inclusion rate and levels of C15:0 and C17:0 in the egg yolk is positive evidence that this effect is due to the inclusion of alfalfa-fed BSF dry larvae in the feed. Revisiting the data on FAs in BSF dry larvae feed (Table 3) shows that both C:15 and C:17 are present at levels of 1–1.4% of the total FAs in BSF. These values are close to the levels of C:15 and C:17 in milk [57,58], and not to previously reported amounts for BSF. Higher levels of C:15 and C:17 FAs in BSF larvae might be explained by the diet components and conditions (temperature in the diet, duration of fermentation processes). Larvae were reared on a fiber-rich diet (5% alfalfa) that stimulates pre-oral and gut fermentation processes with the production of short-chain fatty acids (SCFAs), resulting in increased C:15 and C:17 levels. Once BSF whole dry larvae are part of hens’ diet and ingested, C:15 and C:17 might be directly absorbed by hens’ gut epithelial cells. Furthermore, chitin, as part of a BSF-based diet, may stimulate the synthesis of SCFAs [59] presenting an additional source for C:15 and C:17 production. Future studies will elaborate on these findings and draw a strategy for egg enrichment with C:15 and C:17, which might add value to eggs produced from BSF-fed hens. Regarding the unsaturated FAs, the experimental diets had no effect on the total amounts of MUFAs and PUFAs in the egg yolk, although some responses of individual FAs were observed, especially in the 6% and 9% BSF experimental groups (Table 6).

## 5. Conclusions

The study demonstrated that BSF-based diets modulated egg nutritional parameters without compromising hens’ performance. The inclusion of BSF whole dry larvae in hens’ diet enhanced yolk color and increased yolk carotenoids such as astaxanthin, whereas β-carotene suggested a threshold or plateau responses at the higher inclusion levels.

The study also provided evidence that BSF inclusion enriched the yolk with α-tocopherol (33–37%) in the 6% and 9% BSF-fed groups. Dose–response studies of BSF inclusion rates (3%, 6%, 9%) provided an additional reaffirmation of the results and suggested 6% as the most effective in terms of response and cost.

The study also demonstrated a positive correlation between BSF inclusion rates in hens’ diets and the levels of pentadecanoic acid (C15:0) and heptadecanoic acid (C:17) in the egg yolks. Recent reports on these two odd-chain FAs suggest their crucial role in cell health and longevity. Thus, the study proposes new parameters for egg enrichment through BSF-based diets that may have a positive impact on human health.

Overall, the study showed that the nutritional power of feed comprising BSF whole dry larvae lies in its complex matrix, which can simultaneously modulate multiple aspects of egg quality. Further research is required to optimize BSF production systems and feeding strategies that maximize nutrient transfer efficiency while supporting sustainable poultry production.

## Figures and Tables

**Table 1:** Proximate composition of alfalfa-fed BSF larvae (%/DM).

Component	Content %
Protein	41.65
Total fat	21.85
Carbohydrates	5.61
Sugars	1.25
Fibers	17.3
Ash	5.66
Na	0.11
Ca	0.30
Energy, (kcal/100 g)	419.95

**Table 2:** Composition of the control and the experimental diets.

Item	Control	Treatment Diets (% BSF)		
		3	6	9
Maize	38.000	38.000	38.000	38.000
Wheat	21.000	22.000	23.000	22.500
Sunflower meal	14.500	13.000	13.500	14.535
Soybean meal (45.8% CP)	12.000	10.500	7.450	4.250
BSF dry larvae (41.65% CP)	-	3.000	6.000	9.000
L-Lysine	0.215	0.105	0.015	-
DL-Methionine	0.100	0.110	0.100	0.080
Salt	0.250	0.250	0.250	0.250
Sodium bicarbonate	0.200	0.200	0.200	0.200
Dicalcium phosphate	1.000	1.000	1.050	1.100
Limestone	9.150	9.150	9.000	9.100
Premix ^1^	0.200	0.200	0.200	0.200
Sal Curb	0.200	0.200	0.200	0.200
Alkozel	0.010	0.010	0.010	0.010
Hostazym X15000	0.010	0.010	0.010	0.010
OptiPhos 5000	0.003	0.003	0.003	0.003
And Ox EU	0.012	0.012	0.012	0.012
Sunflower oil	3.100	2.200	0.950	0.500
MTox+	0.050	0.050	0.050	0.050
Proximate composition				
Metabolizable energy (Kcal/kg)	2789	2796	2796	2805
Crude protein,%	16.42	16.86	17.02	17.25
Crude fat, %	4.15	4.03	4.12	4.63
Crude fiber, %	4.82	4.34	4.04	4.53
Ash (%)	10.13	9.88	11.08	9.78
Lysine *, %	0.84	0.86	0.85	0.86
Methionine *, %	0.40	0.40	0.40	0.40
Methionine + Cystine *, %	0.71	0.72	0.73	0.70
Threonine *, %	0.56	0.54	0.52	0.50
Tryptophane *, %	0.18	0.16	0.15	0.14
Na *, %	0.18	0.18	0.18	0.18
Cl *, %	0.20	0.20	0.20	0.20
Ca *, %	3.65	3.65	3.60	3.65
Available P *, %	0.35	0.35	0.35	0.35

^1^ The premix provided the following per kilogram of diet: Vitamin A-10,000 IU; Vitamin D—3000 IU; Vitamin E—20 mg; Vitamin K—3 mg; Vitamin B_1_—2 mg; Vitamin, B_2_—5 mg; Vitamin B_6_—5 mg; Vitamin B_12_—0.016 mg; Nicotinic acid—30 mg; Pantothenic acid—12 mg; Folic acid—0.75 mg; Biotin—0.05 mg; Calcium—400 mg; Iron—50 mg; Manganese—100 mg; Zinc—80 mg; Copper—8 mg; Iodine—1 mg; Selenium—0.2 mg. * Calculated values.

**Table 3:** Fatty acid composition (% from total FA) of alfalfa-fed BSF dry larvae (n = 3, SD±).

SFA	Mean	SD	MUFA	Mean	SD	PUFA	Mean	SD
C10:0	2.24	0.11	C14:1	1.03	0.1	C18:2n6c	6.4	0.22
C12:0	39.2	1.55	C16:1	1.43	0.04	C18:3n6	nd	nd
C14:0	6.27	0.15	C17:1	nd	nd	C18:3n3	nd	nd
C15:0	1.02	0.06	C18:1n9t	nd	nd	C20:2	nd	nd
C16:0	15.56	0.24	C18:1n9c	15.34	0.52	C20:3n6	nd	nd
C17:0	1.36	0.08	C20:1	0.97	0.07	C20:3n3	nd	nd
C18:0	3.55	0.16	C22:1	0.9	0.08	C20:4n6	nd	nd
C20:0	1.84	0.15	C24:1	nd	nd	C22:2	1.1	0.14
C21:0	tr					C20:5n3	nd	nd
C22:0	1.8	0.15				C22:6n3	nd	nd
C23:0	nd							
C24:0	nd							
Total	72.83	0.58	Total	19.67	0.54	Total	7.5	0.32

SFA: saturated fatty acids; MUFA: monounsaturated fatty acids; PUFA: polyunsaturated fatty acids; n-3: omega-3; n-6: omega-6; tr—trace amounts are <0.1% of total fatty acids; nd—not detected.

**Table 4:** Average performance parameters of hens for the period of 4 weeks (n = 5, SE±).

Parameters	Control	Treatment Diets (% BSF)			SEM	*p*- Value	Contrast	
		3	6	9			Linear	Quadr.
Feed intake g/h/d	120.96 ^a^	119.64 ^a^	117.02 ^ab^	114.90 ^b^	1.08	0.004	0.000	0.809
Egg production, %	82.15	84.33	82.80	81.75	1.70	0.723	0.724	0.355
FCR (g/g) *	2.43	2.33	2.36	2.34	0.05	0.429	0.429	0.390
Egg weight, g	60.93	61.21	60.34	60.48	0.42	0.449	0.252	0.867
Egg mass g/h/d **	50.04	51.61	49.94	49.45	0.99	0.465	0.447	0.312

* FCR: Feed-to-egg conversion ratio = average daily feed intake/egg mass; ** Egg mass: Egg weight × egg production/100 (g). ^a,b^ Values with different superscripts in the same row are significantly different (*p* < 0.05); SEM, standard error of the means.

**Table 5:** External and internal quality parameters of eggs produced by the control and experimental groups of hens, (n = 5, SEM±).

Parameters	Control	Treatment Diets (% BSF)			SEM	*p*- Value	Contrast	
		3	6	9			Linear	Quadr.
Egg weight (g)	61.00	59.82	59.39	60.49	0.65	0.300	0.504	0.085
Shape index (%)	77.15	77.47	77.14	77.67	0.37	0.687	0.457	0.777
Shell (%)	9.27	8.91	9.07	9.31	0.12	0.104	0.610	0.018
Albumen (%)	62.28	62.17	62.05	61.44	0.38	0.399	0.116	0.512
Yolk (%)	28.45	28.92	28.87	29.26	0.35	0.439	0.130	0.907
Yolk color units	5.88 ^b^	6.83 ^ab^	7.24 ^a^	7.45 ^a^	0.25	<0.001	<0.001	0.152
Haugh units	81.60	82.10	83.27	78.94	1.14	0.054	0.181	0.037
Shell thickness (mm)	0.339 ^ab^	0.337 ^ab^	0.330 ^b^	0.351 ^a^	0.004	0.008	0.105	0.011

^a,b^ Values with different superscripts in the same row are significantly different (*p* < 0.05); SEM, standard error of the means.

**Table 6:** Fatty acids composition (% of total FA) of eggs from the control and experimental groups (n = 3, SEM±).

Fatty Acids	Control	Treatment Diets (% BSF)			SEM	*p*- Value	Contrast	
		3	6	9			Linear	Quadr.
SFA								
C12:0	nd	nd	nd	nd	-	-	-	-
C14:0	1.52 ^b^	1.82 ^bc^	2.19 ^ac^	2.61 ^a^	0.09	0.004	0.001	0.538
C15:0	0.79	0.94	1.18	1.19	0.09	0.081	0.022	0.486
C16:0	21.98	22.39	22.19	21.37	0.52	0.591	0.431	0.304
C17:0	0.85	1.03	1.26	1.28	0.09	0.080	0.021	0.461
C18:0	7.11 ^b^	7.45 ^a^	7.39 ^ab^	7.62 ^a^	0.06	0.016	0.005	0.383
C20:0	1.65	1.90	2.39	2.43	0.16	0.066	0.018	0.553
Total	34.79	36.86	37.14	38.66	0.69	0.148	0.043	0.712
MUFA								
C14:1	0.78 ^b^	0.94 ^ab^	1.16 ^a^	1.24 ^a^	0.06	0.020	0.005	0.557
C16:1	3.22	3.30	3.36	3.51	0.06	0.105	0.027	0.647
C17:1	0.90	1.03	0.61	1.29	0.31	0.550	0.626	0.431
C18:1n-9	38.50	35.18	34.75	32.32	1.32	0.119	0.033	0.755
C20:1	0.99 ^b^	1.09 ^ab^	1.33 ^ab^	1.36 ^a^	0.07	0.039	0.010	0.626
Total	44.36	41.54	41.41	39.70	1.26	0.213	0.066	0.681
PUFA								
C18:2n-6	12.26	13.23	11.78	11.47	0.48	0.190	0.148	0.252
C20:2	0.98	1.14	1.43	1.48	0.11	0.075	0.019	0.634
C20:3n-3	1.42	1.09	1.75	1.77	0.20	0.194	0.130	0.441
C20:4n-6	2.15 ^ab^	2.10 ^b^	2.47 ^a^	2.47 ^a^	0.07	0.029	0.011	0.752
C22:2	1.02	1.13	0.92	1.01	0.22	0.925	0.822	0.950
C20:5n-3	0.94	1.08	1.40	1.37	0.11	0.106	0.032	0.490
C22:6n-3	1.45	1.45	1.71	1.72	0.08	0.119	0.041	0.953
Total	20.86	21.61	21.46	21.64	0.74	0.860	0.539	0.720

SFA: saturated fatty acids; MUFA: monounsaturated fatty acids; PUFA: polyunsaturated fatty acids; n-3: omega-3; n-6: omega-6; tr—trace amounts are <0.1% of total fatty acids; nd—not detected. ^a,b,c^ Values with different superscripts in the same row are significantly different (*p* < 0.05); SEM, standard error of the means.

## Data Availability

The original contributions presented in the study are included in the article. Further inquiries can be directed to the corresponding author.

## Abbreviations

BSF	Black soldier fly
LDL	Low-density lipoprotein
HDL	High-density lipoprotein
VLDL	Very low-density lipoprotein
FA	Fatty acid
SFA	Saturated fatty acid
MUFA	Monounsaturated fatty acid
PUFA	Polyunsaturated fatty acid
DM	Dry matter
FCR	Feed to egg conversion ratio
SEM	Standard error of the means
